# Evaluating the effectiveness of antiseptic barrier caps in reducing central line-associated bloodstream infections in the adult ICU

**DOI:** 10.1017/ash.2024.462

**Published:** 2024-12-30

**Authors:** Febeena Hussain, Rajalakshmi Ananthanarayanan, Vettakkara Kandy Muhammed Niyas

**Affiliations:** Department of Infectious Diseases, KIMS: KIMSHEALTH, Thiruvananthapuram, KL, India

## Dear Editor,

Central line-associated bloodstream infections (CLABSIs) are significant device-associated infections that increase hospital length of stay (LOS) and can result in death.^1^ Preventing CLABSIs involves a set of practices including the disinfection of central line access ports, which can be done manually with alcohol swabs or by using disinfection caps.^2^ We performed a retrospective analysis to evaluate the effectiveness of antiseptic barrier caps, Curos® by 3M (ABC) in reducing CLABSIs in medical and surgical Intensive Care Units (ICUs).

A retrospective Electronic Medical Records analysis was conducted for patients with central lines admitted to three adult ICUs—two surgical (cardiac surgery ICU and neurosurgery ICU) and one medical ICU. Before implementing ABC, staff received education and training as per guidelines and manufacturer recommendations. Further audits on usage and retraining were done to ensure compliance with ABC use. Other aspects of the CLABSI prevention bundle remained unchanged. The study period differed between ICUs due to staggered implementation. For the cardiac and neurosurgery ICUs, the pre-implementation period was from May 2021 to November 2022, and the post-implementation period was from December 2022 to June 2024. In the medical ICU, the pre-implementation period was from May 2022 to May 2023, and the post-implementation period was from June 2023 to June 2024. The nursing lead ensured compliance with ABC usage, monitored by the number of accesses and ABC use in each nursing shift. CLABSI was defined according to CDC and National Healthcare Safety Network (NHSN) criteria. Data on patients with central venous catheters (CVCs) were collected from individual ICU records as part of ongoing active surveillance by the hospital infection control department. The primary outcome assessed was the CLABSI rate per 1000 catheter days.

During the pre-implementation period, 5965 CVC days were calculated from the ICUs (medical, cardiac surgery, and neurosurgery), and during the post-implementation period, 8059 CVC days were calculated. Before implementing ABC, 26 patients developed CLABSI (medical ICU: 14, cardiac surgery ICU: 7, neurosurgery ICU: 5). After implementation, 26 patients developed CLABSI (medical ICU: 15, cardiac surgery ICU: 4, neurosurgery ICU: 7). Device utilization rates were 0.39 before and 0.43 after ABC implementation, showing comparable use. The CLABSI rate pre-implementation was 4.4 and post-implementation was 3.23, demonstrating a decline in the CLABSI rate. The average additional days of hospital stay for a patient with CLABSI is around 7 days, and by preventing CLABSI, this extra LOS is saved. The antibiotic costs for the treatment of CLABSI vary based on the organism, antibiotic resistance pattern, and duration of therapy, which was not looked at in this study and is in scope for future prospective studies.

The incidence data from the NHSN and the International Nosocomial Infection Control Consortium indicate a significant burden of central line-associated bloodstream infections (CLABSI) in healthcare settings.^3,4^ Recent practice recommendations by the Society for Healthcare Epidemiology of America (SHEA), Infectious Diseases Society of America, and Association for Professionals in Infection Control and Epidemiology (2022 update) outline practices to be followed before, during, and after insertion to further reduce the incidence of CLABSI.^5^ Despite being supported by high-level evidence, antiseptic-containing caps remain an “additional practice” because they are not considered superior to manual disinfection, an essential practice.

During monthly surveillance in our hospital, it was noted that our CLABSI rate was increasing compared to previous internal benchmarks. This increase was initially thought to be a consequence of COVID-19 pandemic-related inadequate compliance with infection control practices and high nurse attrition. CLABSI bundle process audits revealed that “hub the rub” was the most commonly missed or inadequately performed step, even after multiple training and feedback sessions. As an additional measure to reduce CLABSI rates, we introduced ABC in selected surgical and medical ICUs. We noted a reduction in CLABSI rates in all ICUs during the post-implementation period.

Several studies have shown the benefit of ABC in reducing CLABSI. Some studies have shown a cost-benefit. Tejada et al. conducted a systematic review and meta-analysis and found that using antiseptic barrier caps significantly reduces CLABSI rates, especially in ICU patients. The findings suggest antiseptic caps are cost-effective, with median savings of $21,890 per CLABSI.^6^ Cruz-Aguilar et al. demonstrated a significant reduction in CLABSI incidence rates per 1000-line days, with the ABC group showing lower rates (10.38) compared to the control group (15.28).^7^ Helder et al. evaluated antiseptic barrier caps in neonatal and pediatric ICUs, showing a 22% reduction in CLABSI rates from 3.15 to 2.35 per 1000 catheter days, though not statistically significant (*P* = .368). High protocol adherence was noted, particularly in NICUs (95.2%), with a more pronounced effect in the neonatal population.^8^ These studies highlight the potential of antiseptic barrier caps in reducing CLABSI rates and emphasize the importance of adherence, warranting further research to confirm their efficacy across different settings.

In our study, using antiseptic barrier caps as an additional approach appears to reduce the rate of CLABSIs in adult ICUs, as indicated by the lower CLABSI rate post-implementation. Continuous compliance and monitoring are essential for maintaining these results.
